# Red Blood Cell Membrane Conductance in Hereditary Haemolytic Anaemias

**DOI:** 10.3389/fphys.2019.00386

**Published:** 2019-04-16

**Authors:** Polina Petkova-Kirova, Laura Hertz, Jens Danielczok, Rick Huisjes, Asya Makhro, Anna Bogdanova, Maria del Mar Mañú-Pereira, Joan-Lluis Vives Corrons, Richard van Wijk, Lars Kaestner

**Affiliations:** ^1^Institute of Molecular Cell Biology, Saarland University, Homburg, Germany; ^2^Theoretical Medicine and Biosciences, Saarland University, Homburg, Germany; ^3^Experimental Physics, Saarland University, Saarbrücken, Germany; ^4^Department of Clinical Chemistry & Haematology, University Medical Center Utrecht, Utrecht, Netherlands; ^5^Red Blood Cell Research Group, Institute of Veterinary Physiology, Vetsuisse Faculty, Zurich Center for Integrative Human Physiology (ZIHP), University of Zürich, Zurich, Switzerland; ^6^Vall d’Hebron Research Institute, Vall d’Hebron University Hospital, Barcelona, Spain; ^7^Red Blood Cell Defects and Hematopoietic Disorders Unit, Josep Carreras Leukaemia Research Institute, Barcelona, Spain

**Keywords:** haemolytic anemia, patch-clamp, electrophysiology, hereditary spherocytosis, hereditary xerocytosis

## Abstract

Congenital haemolytic anaemias are inherited disorders caused by red blood cell membrane and cytoskeletal protein defects, deviant hemoglobin synthesis and metabolic enzyme deficiencies. In many cases, although the causing mutation might be known, the pathophysiology and the connection between the particular mutation and the symptoms of the disease are not completely understood. Thus effective treatment is lagging behind. As in many cases abnormal red blood cell cation content and cation leaks go along with the disease, by direct electrophysiological measurements of the general conductance of red blood cells, we aimed to assess if changes in the membrane conductance could be a possible cause. We recorded whole-cell currents from 29 patients with different types of congenital haemolytic anaemias: 14 with hereditary spherocytosis due to mutations in α-spectrin, β-spectrin, ankyrin and band 3 protein; 6 patients with hereditary xerocytosis due to mutations in Piezo1; 6 patients with enzymatic disorders (3 patients with glucose-6-phosphate dehydrogenase deficiency, 1 patient with pyruvate kinase deficiency, 1 patient with glutamate-cysteine ligase deficiency and 1 patient with glutathione reductase deficiency), 1 patient with β-thalassemia and 2 patients, carriers of several mutations and a complex genotype. While the patients with β-thalassemia and metabolic enzyme deficiencies showed no changes in their membrane conductance, the patients with hereditary spherocytosis and hereditary xerocytosis showed largely variable results depending on the underlying mutation.

## Introduction

Haemolytic anaemias, characterized by the abnormal breakdown of red blood cells (RBCs), could be either acquired or inherited. The latter are a diverse group of diseases that could be classified based on the affected RBC component into membranopathies, haemoglobinopathies, and enzymopathies (Dhaliwal et al., 2004). Membranopathies are presented by hereditary spherocytosis (HS), hereditary elliptocytosis (HE) and its aggravated form pyropoikilocytosis (HPP) with defective structural membrane and cytoskeletal proteins (Iolascon et al., 2003) and by the largely heterogeneous group of stomatocytosis divided in a most general way, but not exhaustively, into overhydrated stomatocytosis (OHSt), cryohydrocytosis (CHC) and some types of familial pseudohyperkalaemia (FP) (overhydrated RBCs) and dehydrated stomatocytosis (DHSt) (hereditary xerocytosis (HX)) (dehydrated RBCs) with defective ion channels or transporters (Iolascon et al., 2003; Bruce et al., 2009). Haemoglobinopathies are presented by β-thalassemia (Cao and Galanello, 2010) and sickle cell disease (Ware et al., 2017) with defective hemoglobin and enzymopathies are presented most commonly by glucose-6-phosphate dehydrogenase deficiency (G6PD) (Luzzatto et al., 2016) and pyruvate kinase deficiency (PKD) (Zanella et al., 2005) but also by glutamate-cysteine ligase (γ-glutamylcysteine synthetase) (GCL) deficiency (Ristoff and Larsson, 1998) and glutathione reductase deficiency (van Zwieten et al., 2014). Although much is known so far, especially regarding the defective genes related to hereditary haemolytic anaemias, there are still questions, whose answers would lead to a much better understanding of the disease and possibly to a more effective treatment. A recurrent issue is if a changed membrane conductance, resulting from primary mutated channels or secondary adapted ones, does contribute to the various phenotypes. Most of the research has been done on membranopathies, understandable, primarily on the ones linked to mutations in ion channels or transporters such as band 3 protein, Rh-associated glycoprotein (RhAG), the glucose transporter GLUT1, Piezo1 and the Gardos channel (KCNN4) and accompanied by abnormal RBC cation content and disrupted volume homeostasis (Badens and Guizouarn, 2016). However, although on most occasions, the RBC cation content linked to the particular mutation has been extensively described (e.g., Stewart et al., 2011 for R730C in band 3 protein or Fermo et al., 2017 for R352H in the Gardos channel) and the defective channels, when known, expressed and studied in heterologous systems (e.g., Glogowska et al., 2017 for a number of Piezo1 mutations) with a few exceptions (Stewart et al., 2011; Andolfo et al., 2013; Shmukler et al., 2014; Fermo et al., 2017; Rotordam et al., 2018), direct electrophysiological measurements of membrane conductance in mutated RBCs have been scarce.

Thus, within the CoMMiTMenT project, by direct RBCs electrophysiological measurements in physiological solutions, we aimed to investigate whether RBC membrane conductance changes are accompanying HS due to mutations in the SPTA1 gene (coding for α-spectrin), SPTB (β-spectrin), ANK1 (ankyrin), and SLC4A1 (band 3 protein); HX due to mutations in PIEZO1; enzymatic disorders due to glucose-6-phosphate dehydrogenase deficiency (G6PD), pyruvate kinase deficiency (PKD), glutamate-cysteine ligase (γ-glutamylcysteine synthetase) (GCL) deficiency, glutathione reductase deficiency and β-thalassemia.

## Materials and Methods

### Patients

Patients diagnosed with different types of haemolytic anemia were enrolled in the study after signing an informed consent. Patients’ data were handled anonymously as outlined in the ethics agreements. These agreements were approved by the Medical Ethical Research Board (MERB) of the University Medical Center Utrecht, the Netherlands (UMCU) under reference code 15/426M “Disturbed ion homeostasis in hereditary hemolytic anemia” and by the Ethical Committee of Clinical Investigations of Hospital Clinic, Spain (IDIBAPS) under reference code 2013/8436. Exclusion criteria were erythrocyte transfusion in the past 90 days, age below 3 years and/or bodyweight lower than 18 kg. Blood from healthy control donors was anonymously obtained using the approved medical ethical protocol of 07/125 Mini Donor Dienst, also approved by the MERB of UMCU. The blood of the patient/patients and the healthy donor anti-coagulated in heparin was shipped overnight from the University Medical Center Utrecht (Utrecht, Netherlands) or from Institut d’Investigacions Biomèdiques August Pi i Sunyer/Hospital Clínic de Barcelona (Barcelona, Spain) to Saarland University (Homburg, Germany) without additional cooling as previously tested/simulated (Makhro et al., 2016). All patients included in the study were genetically screened for mutations by next-generation sequencing and diagnosed with the following types of anemia: 14 patients were diagnosed with HS (due to mutations in α-spectrin, β-spectrin, ankyrin and band 3 protein), using golden standard techniques (EMA-binding, osmotic gradient ektacytometry and osmotic fragility test), 6 patients were diagnosed with hereditary xerocytosis (due to mutations in Piezo 1), 6 patients had enzymatic disorders (3 patients with glucose-6-phosphate dehydrogenase deficiency, 1 patient with pyruvate kinase deficiency, 1 patient with glutamate-cysteine ligase deficiency and 1 patient with glutathione reductase deficiency), 1 patient had β-thalassemia and 2 were carriers of several mutations and a complex genotype. The genotype of the patients with HS, HX and of the two patients with several mutations is given in Table 1. The numbering of the patients and the corresponding healthy controls is kept consistent with previous research (Hertz et al., 2017) studying the same patient group.

**Table 1:** Patients overview.

Patient	Clinical presentation	Genotype	Current compared to transportation control	Current compared to general control
**P18.1**	**HS (α-spectrin)**	**c.2755G > T (p.Glu919); α^LELY^**	**Current ↑**	**Current ↑**
**P19.1**	**HS (α-spectrin)**	**c.678G > A (p.Glu227fs); α^LELY^**	**Current ↑ (not statistically significant)**	**Current ↑**
P15.1	HS (α-spectrin)	c.4339-99C > T p.(?)	No change	No change
P20.1	HS (α-spectrin)	c.[4339-99C > T; c.4347G > T] p.[(?; Lys1449Asn)]	No change	No change
P17.1 (splenectomized)	HS (ankyrin)	c.341C > T (p.Pro114Leu)	Current ↓ ^∗^	No change
P17.2	HS (ankyrin)	c.341C > T (p.Pro114Leu)	Current ↓ ^∗^	No change
P11.1	HS (ankyrin)	c.1943delC;c.2042delC (p.Ala648fs;p.Ala681fs)	No change	No change
P13.1	HS (ankyrin)	c.344T > C (p.Leu115Pro)	No change	No change
P13.2	HS (ankyrin)	c.344T > C (p.Leu115Pro)	No change	No change
P14.1	HS (ankyrin)	c.2559-2A > G (splicing)	No change	No change
P10.1	HS (β-spectrin)	c.2470C > T (p.Gln824)	Outward current ↓↓ ^∗^	No change
P21.1	HS (β-spectrin)	c.3449G > A (p.Trp1150)	No change	No change
P12.1	HS (band 3 protein)	c.2348T > A (p.Ile783Asn)	Current ↓	Inward current ↓
P16.1	HS (band 3 protein)	c.2057 + 1G > A (splicing)	No change	No change
**P11.1**	**SPTB**	**c.154delC; p.Arg52fs + RHAG c.808G > A; p.Val270Ile**	**Current ↑**	**Current ↑**
**P22.1**	**SPTA1**	**c.460_462dupTTG; p.Leu154dup + PKLR c.1687G > A; p.Gly563Ser + del3.7Kb HBA**	**Current ↑**	**Current ↑**
Family 1				
P51.3	HX	c. 7367G > A p.Arg2456His	No change	No change
P51.4	HX	c. 7367G > A p.Arg2456His	No change	No change
Family 2				
P53.3	HX	c. 6262C > G, p. Arg2088Gly	No change	No change
P53.2	HX	c. 6262C > G, p. Arg2088Gly	No change	No change
Family 3				
P50.2	HX	c.1276T > C p. Cys426Arg	No change	Outward current ↑ ^∗^
Family 4				
**P52.1**	**HX**	**c.7483_7488dupCTGGAG p.2495_2496dupLeuGlu**	**Current ↓**	**Current ↓**
P40.1	G6PD		No change	No change
P41.1	G6PD		No change	No change
P42.1	G6PD		No change	No change
P43.1	GCLD		No change	No change
P44.1	GRD		No change	No change
P45.1	PKD		No change	No change
P60.1	β-thalassemia		No change	No change


*An upward arrow indicates an increase in current and a downward arrow indicates a decrease in current compared to the respective control. Two arrows label an extreme change in the designated direction. Patients that show a change in current compared to both the transportation and a general control are given in bold. Asterisks mark a deviation commented on in the main text. HS, hereditary spherocytosis; SPTB, β-spectrin; SPTA1, α-spectrin; HX, hereditary xerocytosis; GCLD, glutamate-cysteine ligase deficiency; GRD, glutathione reductase deficiency; PKD, pyruvate kinase deficiency.*

### Patch Clamp Analysis

Patch-clamp whole-cell measurements were performed with a NPC-16 Patchliner (Nanion Technologies, Munich, Germany) as previously described (Petkova-Kirova et al., 2018). Briefly, the resistance of the chips was between 5 and 8 MΩ with internal and external solutions as follows (in mM): KCl 70, KF 70, NaCl 10, HEPES 10, EGTA 3, CaCl_2_ 1.2 to give free [Ca^2+^]_i_ = 120 nM, pH = 7.2 adjusted with KOH (internal) and NaCl 140, KCl 4, MgCl_2_ 5, CaCl_2_ 2, D-glucose 5, HEPES 10, pH = 7.3 adjusted with NaOH (external). Gigaseals were considered successful if exceeding 5 GΩ. Gigaseal formation was facilitated by the use of a seal enhancing solution as recommended by the Patchliner manufacturer and containing (in mM): NaCl 80, KCl 3, MgCl_2_ 10, CaCl_2_ 35, HEPES 10, pH = 7.3 adjusted with NaOH. Whole-cell configuration was achieved by negative pressure suction pulses between -45 mbar and -150 mbar and its formation judged by the appearance of sharp capacitive transients. Whole-cell patch-clamp recordings were conducted at room temperature using voltage steps from -100 to 100 mV for 500 ms in 20 mV increments at 5 s intervals, the holding potential being set at -30 mV. Data are presented as current density (current divided by capacitance, the latter estimated at the time of attaining the whole-cell configuration and by using a short test pulse of 10 mV, 5 ms) and given as means ± SEMs (*n* denotes number of cells and *N* – number of patients). Significant differences are determined based on an unpaired *t*-test and Welch’s correction for unequal variances, when needed, with ^∗^ denoting *p* < 0.05.

## Results

Whole-cell patch clamp measurements were performed to assess possible differences in the membrane conductance of hereditary anemia patients compared to healthy controls. Regarding controls, we have compared the currents measured from the RBCs of our patients once with their transportation control, i.e., currents measured from the RBCs of a healthy subject, whose blood was delivered together with the blood of the patient, and once with a general, pooled, control, i.e., currents measured from the cells of all healthy subjects delivered throughout the study (Table 1). The rational for this ‘double comparison’ is provided in the discussion. Throughout the whole study, accordingly in the manuscript, the abbreviation “P” stands for patient and “C” stands for a control, healthy subject.

### Hereditary Spherocytosis

**FIGURE 1:**
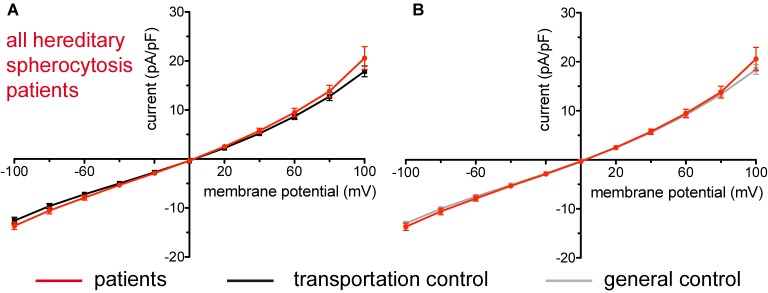
Whole-cell recordings of ion currents from RBCs of healthy donors and HS patients. Compared are the I/V-curves of all HS patients (*N* = 14) with the I/V curves of their transportation controls (*N* = 12) **(A)** and with the I/V curves of all healthy subjects delivered throughout the study (*N* = 27) **(B)**, where *N* denotes number of healthy subjects or HS patients. No changes were observed in capacitance either with the transportation (0.63 pA/pF patients vs. 0.65 pA/pF transportation control; *p* > 0.05) or with the general control (0.63 pA/pF patients vs. 0.69 pA/pF general control; *p* > 0.05). Currents were elicited by voltage steps from –100 to 100 mV for 500 ms in 20 mV increments at *V*_h_ = –30 mV. Data are expressed as mean current density ± SEMs.

**FIGURE 2:**
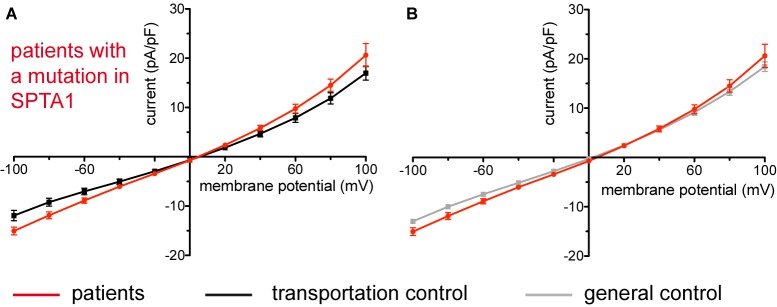
Whole-cell recordings of ion currents from RBCs of healthy donors and HS patients with α-spectrin mutations. Compared are the I/V-curves of all HS patients with α-spectrin mutations (*N* = 4) with the I/V curves of their own transportation controls (*N* = 4) **(A)** and with the I/V curves of all healthy subjects delivered throughout the study (*N* = 27) **(B)**, where *N* denotes the number of healthy subjects or HS patients. No changes were observed in capacitance either with the transportation (0.67 pA/pF patients vs. 0.68 pA/pF control; *p* > 0.05) or with the general control (0.67 pA/pF patients vs. 0.69 pA/pF control; *p* > 0.05). Currents were elicited by voltage steps from –100 to 100 mV for 500 ms in 20 mV increments at *V*_h_ = –30 mV. Data are expressed as mean current density ± SEMs.

**FIGURE 3:**
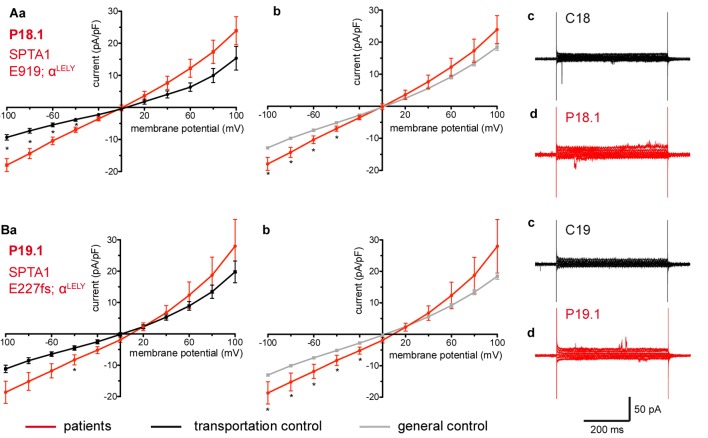
Whole-cell recordings of ion currents from RBCs of healthy donors and HS patients with α-spectrin mutations and carrying at the same time an α^LELY^ allele. Compared are the I/V curves of P18.1 (*n* = 10) with its own transportation control C18 (*n* = 6) **(Aa)** as well as with a general control (*n* = 175) **(Ab)**, where *n* denotes the number of cells from the patient or the controls. As examples raw current traces recorded from the RBCs of a healthy donor (C18), whose blood was delivered together with the blood of P18.1 **(Ac)** and of patient P18.1 **(Ad)** are presented. Capacitances were not any different either with the transportation control (0.59 pA/pF P18.1 vs. 0.74 pA/pF C18; *p* > 0.05) or with the general control (0.59 pA/pF patient vs. 0.69 pA/pF control; *p* > 0.05). Compared are the I/V curves of P19.1 (*n* = 6) with its own transportation control C19 (*n* = 7) **(Ba)** as well as with a general control (*n* = 175) **(Bb)**, where *n* denotes the number of cells from the patient or the controls. As examples raw current traces recorded from the RBCs of a healthy donor (C19), whose blood was delivered together with the blood of P19.1 **(Bc)** and of patient P19.1 **(Bd)** are presented. Capacitances were not any different either with the transportation control (0.66 pA/pF P19.1 vs. 0.58 pA/pF C19; *p* > 0.05) or with the general control (0.66 pA/pF patient vs. 0.69 pA/pF general control; *p* > 0.05). Significant differences are determined based on an unpaired *t*-test with ^∗^ representing *p* < 0.05. Mutations below patients numbers are designated as amino acid substitutions in the respective protein. The label α^LELY^ next to the mutation stands for the presence of an α^LELY^ allele in the corresponding patient.

**FIGURE 4:**
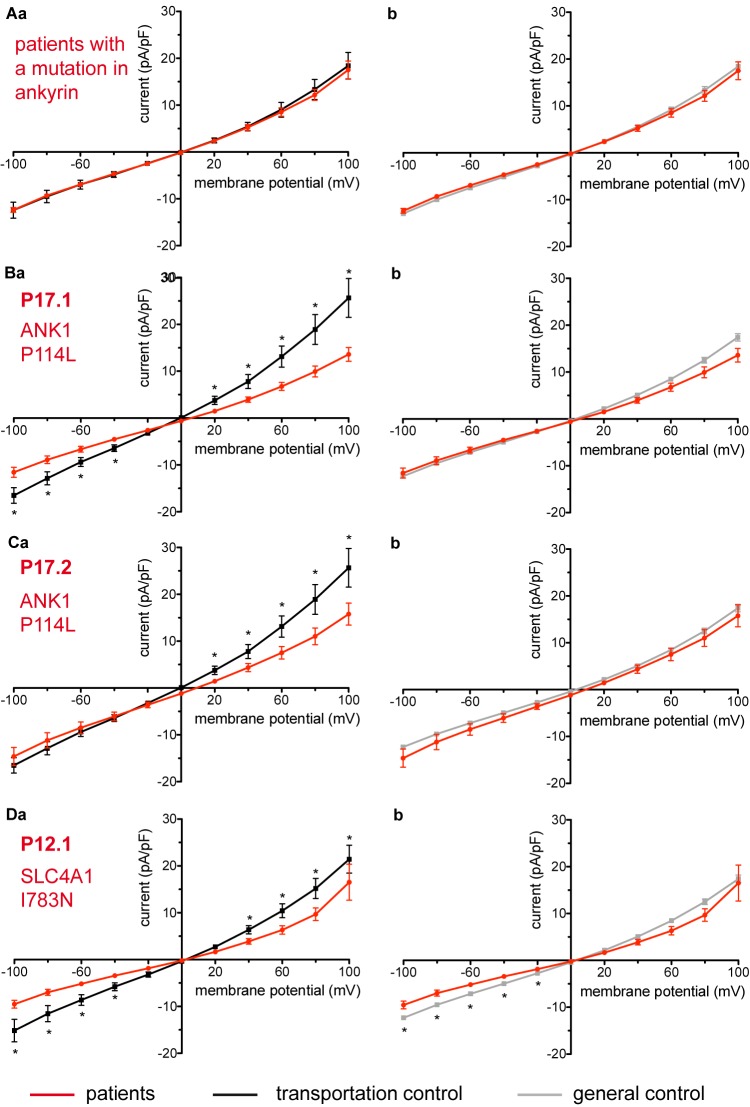
Whole-cell recordings of ion currents from RBCs of healthy donors and HS patients. Whole-cell recordings of ion currents from RBCs of healthy donors and HS patients with ankyrin mutations **(A–C)**. Compared are the I/V-curves of all HS patients with ankyrin mutations (*N* = 6) with the I/V curves of their own transportation controls (*N* = 4) **(Aa)** and with the I/V curves of all healthy subjects delivered throughout the study (*N* = 27) **(Ab)**, where *N* denotes the number of healthy subjects or HS patients. No changes were observed in capacitance either with the transportation (0.67 pA/pF patients vs. 0.63 pA/pF transportation control; *p* > 0.05) or with the general control (0.67 pA/pF patients vs. 0.69 pA/pF general control; *p* > 0.05). Compared are the I/V curves of P17.1 (*n* = 13) and its own transportation control C17 (*n* = 6) **(Ba)** and the I/V curves of P17.2 (*n* = 11) with its own transportation control C17 (*n* = 6) **(Ca)**, where *n* denotes the number of cells from the patient or the control. **(Bb,Cb)** Compare the I/V curve of a general control based on currents recorded from all cells of all healthy subjects delivered throughout the study (*n* = 175) with the I/V curve of P17.1 (*n* = 13) **(Bb)** and P17.2 (*n* = 11) **(Cb)** (*n* denotes number of cells). Both patients 17.1 and 17.2 showed no differences in capacitance either with their transportation or with the general control (0.67 pA/pF P17.1 vs. 0.525 pA/pF C17, *p* > 0.05; 0.67 pA/pF P17.1 vs. 0.69 pA/pF general control, *p* > 0.05; 0.62 pA/pF P17.2 vs. 0.525 pA/pF C17, *p* > 0.05; 0.62 pA/pF P17.2 vs. 0.69 pA/pF general control, *p* > 0.05). However, a difference was uncovered between C17 and the general control (0.525 pA/pF C17 vs. 0.69 pA/pF general control; *p* < 0.05). Whole-cell recordings of ion currents from RBCs of a healthy donor and a HS patient with band 3 protein mutation **(D)**. Compared are the I/V curves of P12.1 (*n* = 17) with its own transportation control C12 (*n* = 7) **(Da)** as well as with a general control (*n* = 175) **(Db)**, where n is the number of cells of the patient or the controls. No changes were observed in capacitance either with the transportation (0.65 pA/pF P12.1 vs. 0.59 pA/pF C12; *p* > 0.05) or with the general control (0.65 pA/pF P12.1 vs. 0.69 pA/pF general control; *p* > 0.05). Currents were elicited by voltage steps from –100 to 100 mV for 500 ms in 20 mV increments at *V*_h_ = –30 mV. Data are expressed as mean current density ± SEMs. Significant differences are determined based on an unpaired *t*-test with ^∗^ representing *p* < 0.05. Mutations below patients numbers are designated as amino acid substitutions in the respective protein.

**FIGURE 5:**
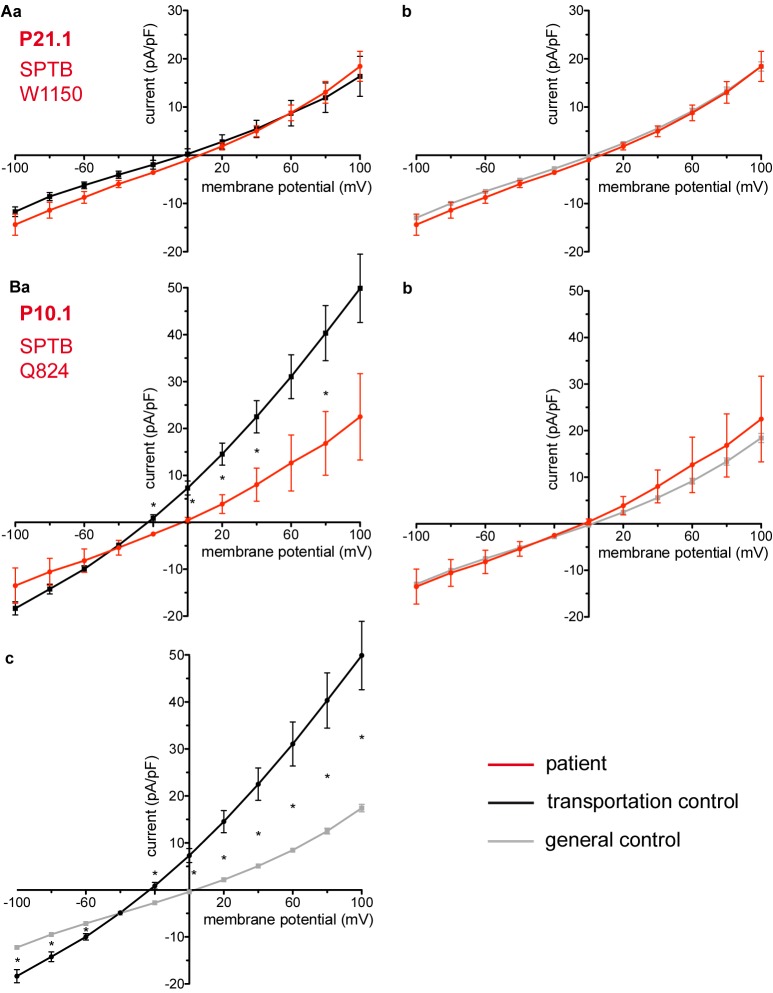
Whole-cell recordings of ion currents from RBCs of healthy donors and HS patients. Whole-cell recordings of ion currents from RBCs of healthy donors and HS patients with β-spectrin mutations **(A,Ba,b)**. Compared are the I/V curves of P21.1 (*n* = 8) with its own transportation control C21 (*n* = 5) **(Aa)** as well as with a general control (*n* = 175) **(Ab)**. Compared are the I/V curves of P10.1 (*n* = 3) with its own transportation control C10 (*n* = 6) **(Ba)** as well as with a general control (*n* = 175) **(Bb)**. **(Bc)** Compares the I/V curve of the transportation control of P10.1, C10 (*n* = 6) with a general control (*n* = 175), where *n* denotes the number of cells. Currents were elicited by voltage steps from –100 to 100 mV for 500 ms in 20 mV increments at *V*_h_ = –30 mV. Data are expressed as mean current density ± SEMs. Significant differences are determined based on an unpaired *t*-test with ^∗^ representing *p* < 0.05. Mutations below patients numbers are designated as amino acid substitutions in the respective protein.

Studied were patients with pathogenic mutations in *SPTA1* (α-spectrin) (4 patients: P15.1, P18.1, P19.1, P20.1), *SPTB* (β-spectrin) (2 patients: P10.1 and P21.1), *ANK1* (ankyrin) (6 patients: P11.1, P13.1, P13.2, P14.1, P17.1, and P17.2), and *SLC4A1* (band 3 protein) (2 patients: P12.1 and P16.1), whose blood was delivered and, respectively, recorded from together with the blood of a healthy subject (transportation control). While no changes in the membrane conductance, nor in membrane capacitance (0.69 pA/pF general control vs. 0.63 pA/pF patients, *p* > 0.05; 0.65 pA/pF transportation control vs. 0.63 pA/pF patients; *p* > 0.05) were revealed with patients taken altogether (Figure 1), differences were observed in certain patients’ groups as well as linked to particular mutations Figure 3–5). Thus patients with mutations in SPTA1 (4 patients), showed no significant differences compared to healthy controls delivered at the same days (4 healthy subjects) (Figure 2A) or compared to a control pooled over all healthy subjects included in the study (27 healthy subjects) (Figure 2B). Capacitances were not different either (0.67 pA/pF patients vs. 0.68 pA/pF transportation control, *p* > 0.05; 0.67 pA/pF patients vs. 0.69 pA/pF general control; *p* > 0.05). However, the two patients, heterozygous for the *SPTA1* mutation and carrying at the same time an α^LELY^ allele showed an increase in their inward current (Figure 3). Figure 3Aa,Ba consider the particular *SPTA1* α^LELY^ patients [patient P18.1 (10 cells) and patient P19.1 (6 cells), respectively] vs. their transportation controls [C18 (6 cells) and C19 (7 cells), respectively]. Figure 3Ab,Bb compare the particular *SPTA1* α^LELY^ patients [P18.1 (10 cells) and P19.1 (6 cells) vs. a control pooled over all the cells of all healthy subjects included in the study (175 cells)]. Figure 3Ac,Ad,Bc,Bd present raw current traces recorded from the RBCs of a healthy subject (Figure Ac,Bc), P18.1 (Figure 3Ad), and P19.1 (Figure 3Bd). None of the two patients showed any difference in capacitance compared with the general or with its transportation control (0.59 pA/pF P18.1 vs. 0.74 pA/pF C18, *p* > 0.05; 0.59 pA/pF P18.1 vs. 0.69 pA/pF general control, *p* > 0.05; 0.66 pA/pF P19.1 vs. 0.58 pA/pF C19, *p* > 0.05; 0.66 pA/pF P19.1 vs. 0.69 pA/pF general control, *p* > 0.05). Furthermore, while HS patients with underlying defects in *ANK1* (6 patients) showed no significant differences neither in their currents, nor in their capacitances (0.67 pA/pF patients vs. 0.63 pA/pF transportation control, *p* > 0.05; 0.67 pA/pF patients vs. 0.69 pA/pF general control; *p* > 0.05) compared to the control group [Figure 4Aa considered are the control healthy subjects delivered together with the patients (4 healthy subjects) and Figure 4Ab considered are all healthy subjects included in the study (27 healthy subjects)], there was a family of patients P17.1 (splenectomized) and P17.2 (with spleen) in whom an *ANK1* mutation [c.341C > T (p.Pro114Leu)] was associated with a decreased membrane conductance when compared to their own controls (Figure 4Ba,Ca, respectively) but not when compared to the pooled control of all healthy cells (Figure 4Bb,Cb, respectively). Comparison of the capacitances of P17.1 and P17.2 with their controls as well as the capacitance of C17 with the general control is as follows: 0.67 pA/pF P17.1 vs. 0.525 pA/pF C17, *p* > 0.05; 0.67 pA/pF P17.1 vs. 0.69 pA/pF general control, *p* > 0.05; 0.62 pA/pF P17.2 vs. 0.525 pA/pF C17, *p* > 0.05; 0.62 pA/pF P17.2 vs. 0.69 pA/pF general control, *p* > 0.05; 0.525 pA/pF C17 vs. 0.69 pA/pF general control; *p* < 0.05. Moreover a patient with a band 3 protein mutation [SLC4A1 (2348T > A, Ile783Asn), P12.1 showed a decreased current compared to its own, transportation, control (C12) and to a pooled general control (Figure 4Da,b, respectively)]. No difference was found when the capacitance of the patient was compared with that of the general or the transportation control (0.65 pA/pF P12.1 vs. 0.59 pA/pF C12, *p* > 0.05; 0.65 pA/pF P12.1 vs. 0.69 pA/pF general control, *p* > 0.05). Out of the two patients with SPTB mutations (P10.1 and P21.1) (Figure 5A,Ba,b) one patient, P10.1, showed a significantly different conductance compared to its transportation control C10 (Figure 5Ba). However, based on the fact that P10.1 showed no difference with the general control (Figure 5Bb) and that P21.1 showed no difference either with its transportation control (Figure 5Aa) or with the general control (Figure 5Ab) as well as on the fact that C10 is very different from the general, pooled control (an outlier according to the Grubbs’ test) (Figure 5Bc), we conclude that patients with β-spectrin mutations show no changes in their current. Comparison of capacitances of P21.1 and P10.1 with their controls as well as the capacitance of C10 with the general control is as follows: 0.63 pA/pF P21.1 vs. 0.69 pA/pF C21, *p* > 0.05; 0.63 pA/pF P21.1 vs. 0.69 pA/pF general control, *p* > 0.05; 0.68 pA/pF P10.1 vs. 0.82 pA/pF C10, *p* < 0.05; 0.68 pA/pF P10.1 vs. 0.69 pA/pF general control, *p* > 0.05; 0.82 pA/pF C10 vs. 0.69 pA/pF general control; *p* < 0.05. C10 shows a significantly increased capacitance compared to P10.1 as well as to the general control.

**FIGURE 6:**
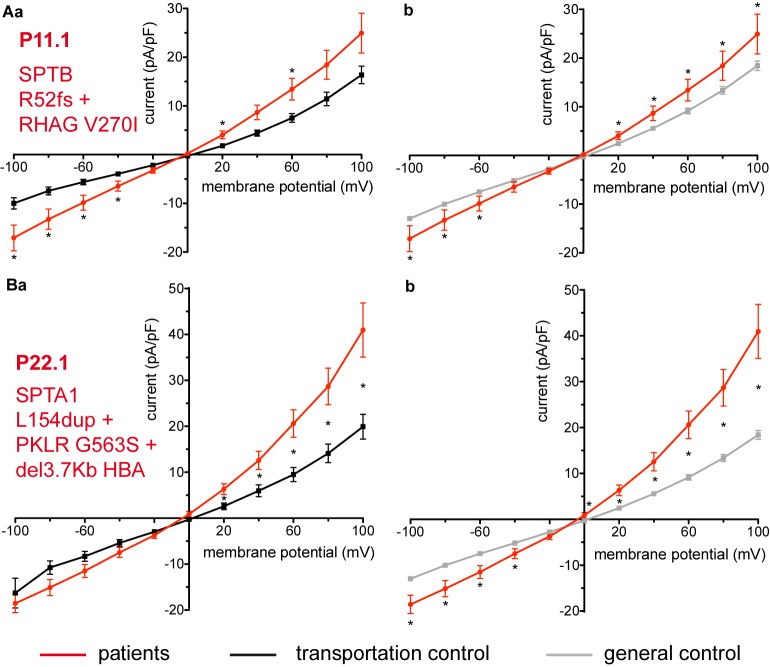
Whole-cell recordings of ion currents from RBCs of healthy donors and patients, carriers of several mutations and a complex genotype. Compared are the I/V curves of P11.1 (*n* = 11) with its own transportation control C11 (*n* = 13) **(Aa)** as well as with a general control (*n* = 175) **(Ab)**. Compared are the I/V curves of P22.1 (*n* = 8) with its own transportation control C22 (*n* = 5) **(Ba)** as well as with a general control (*n* = 175) **(Bb)**, where *n* denotes the number of cells. Currents were elicited by voltage steps from –100 to 100 mV for 500 ms in 20 mV increments at *V*_h_ = –30 mV. Data are expressed as mean current density ± SEMs. Significant differences are determined based on an unpaired *t*-test with ^∗^ representing *p* < 0.05. Mutations below patients numbers are designated as amino acid substitutions in the respective proteins. Additionally to having an α-spectrin mutation P22.1 is PK deficiency carrier as well as an α-thalassemia carrier.

Two additional patients P11.1 and P22.1, carriers of several mutations and a complex genotype (*SPTB* c.154delC; p.Arg52fs + *RHAG* c.808G > A; p.Val270Ile and *SPTA1* c.460_462dupTTG; p.Leu154dup + *PKLR* c.1687G > A; p.Gly563Ser + del3.7Kb *HBA*, respectively) show an increase in their currents (Figure 6A,B, respectively). The capacitances of none of the patients show any difference with their controls (0.59 pA/pF P11.1 vs. 0.66 pA/pF C11, *p* > 0.05; 0.59 pA/pF P11.1 vs. 0.69 pA/pF general control, *p* > 0.05; 0.61 pA/pF P22.1 vs. 0.64 pA/pF C22, *p* > 0.05; 0.61 pA/pF P22.1 vs. 0.69 pA/pF general control, *p* > 0.05).

### Hereditary Xerocytosis

**FIGURE 7:**
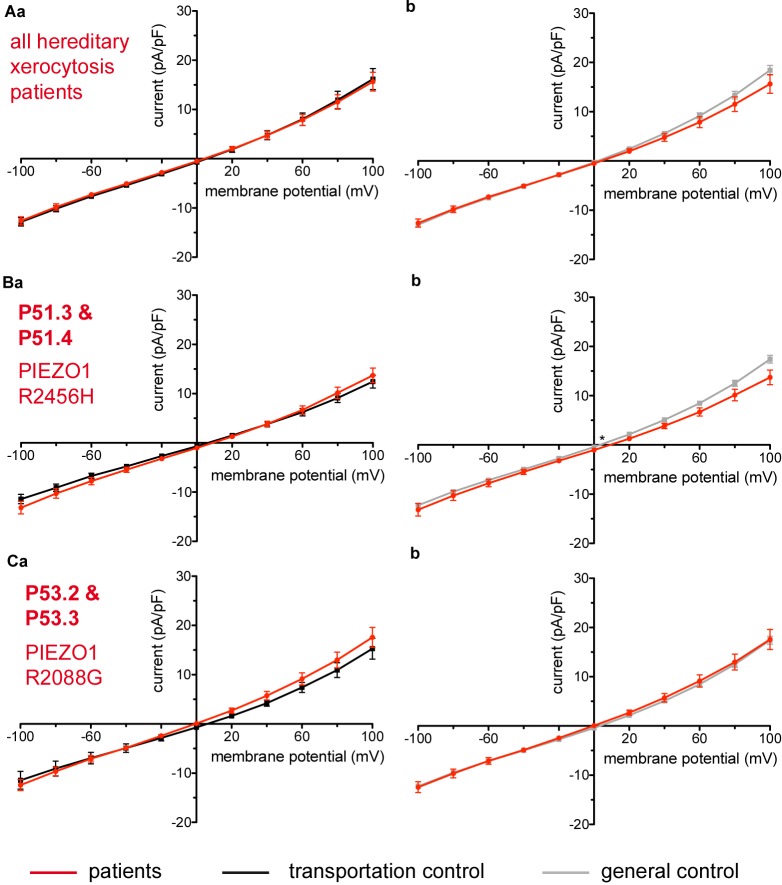
Whole-cell recordings of ion currents from RBCs of healthy donors and HX patients. Compared are the I/V-curves of all HX patients (*N* = 6) with the I/V curves of their own transportation controls (*N* = 4) **(Aa)** and with the I/V curves of all healthy subjects delivered throughout the study (*N* = 27) **(Ab)**, where *N* denotes the number of healthy subjects or HX patients. No changes were observed in capacitance either with the transportation (0.725 pA/pF patients vs. 0.71 pA/pF transportation control; *p* > 0.05) or with the general control (0.725 pA/pF patients vs. 0.69 pA/pF general control; *p* > 0.05). Compared are the I/V curves of P51.3 and P51.4 pooled together (Family 1) (*n* = 22) with their own transportation control C51 (*n* = 7) **(Ba)** and the I/V curves of P53.2 and P53.3 pooled together (Family 2) (*n* = 27) with their own transportation control C53 (*n* = 9) **(Ca)**, where *n* denotes the number of cells from the patients or the controls. **(Bb,Cb)** Compare the I/V curve of a general control based on currents recorded from all cells of all healthy subjects delivered throughout the study (*n* = 175) with the I/V curve of P51.3 and P51.4 pooled together (*n* = 22) **(Bb)** and P53.2 and P53.3 pooled together (*n* = 27) **(Cb)** (*n* denotes number of cells). P51.3 and P51.4 pooled together (Family 1) and P53.2 and P53.3 pooled together (Family 2) did not show a difference in their capacitance compared to the general or transportation control (0.69 pA/pF P51.3 and P51.4 vs. 0.72 pA/pF C51, *p* > 0.05; 0.69 pA/pF P51.3 and P51.4 vs. 0.69 pA/pF general control, *p* > 0.05; 0.75 pA/pF P53.2 and P53.3 vs. 0.71 pA/pF P53, *p* > 0.05; 0.75 pA/pF P53.2 and P53.3 vs. 0.69 pA/pF general control, *p* > 0.05) Currents were elicited by voltage steps from –100 to 100 mV for 500 ms in 20 mV increments at *V*_h_ = –30 mV. Data are expressed as mean current density ± SEMs. Significant differences are determined based on an unpaired *t*-test with ^∗^ representing *p* < 0.05. Mutations below patients numbers are designated as amino acid substitutions in the respective protein.

**FIGURE 8:**
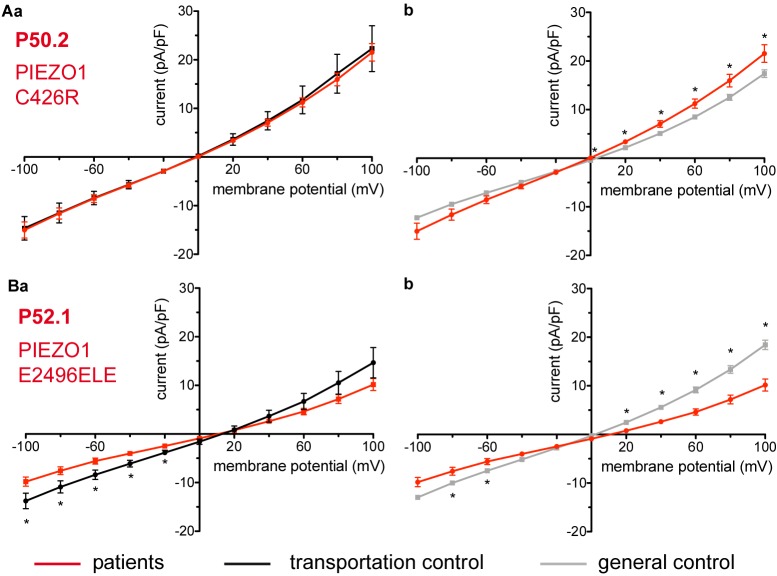
Whole-cell recordings of ion currents from RBCs of healthy donors and HX patients. Compared are the I/V curves of P50.2 (*n* = 14) with its own transportation control C50 (*n* = 7) **(Aa)** as well as with a general control (*n* = 175) **(Ab)**. Comparison of capacitance of P50.2 with control capacitances gave no difference with the general or the transportation control (0.73 pA/pF P50.2 vs. 0.80 pA/pF transportation control; *p* > 0.05; 0.73 pA/pF P50.2 vs. 0.69 pA/pF general control; *p* > 0.05). Compared are the I/V curves of P52.1 (*n* = 20) with its own transportation control C52 (*n* = 11) **(Ba)** as well as with a general control (*n* = 175) **(Bb)**, where n denotes the number of cells. No changes were observed in capacitance either with the transportation (0.75 pA/pF P52.1 vs. 0.62 pA/pF C52; *p* > 0.05) or with the general control (0.75 pA/pF P52.1 vs. 0.69 pA/pF general control; *p* > 0.05). Currents were elicited by voltage steps from –100 to 100 mV for 500 ms in 20 mV increments at *V*_h_ = –30 mV. Data are expressed as mean current density ± SEMs. Significant differences are determined based on an unpaired *t*-test with ^∗^ representing *p* < 0.05. Mutations below patients numbers are designated as amino acid substitutions in the respective protein.

Considered were 4 families with mutations in the PIEZO1 gene (Family 1 with patients P51.3 and P51.4; Family 2 with P53.2 and P53.3; Family 3 with P50.2 and Family 4 with P52.1), whose blood was delivered and recorded from, together with the blood of a healthy subject (transportation control) (C51, C53, C50, and C52, respectively). No changes in the membrane conductance or in the membrane capacitance were revealed with patients taken altogether (Figure 7A) as well as in two families (Family1 (P51.3 and P51.4) and Family 2 (P53.2 and P53.3) compared both to their transportation controls and to a general control (Figure 7Ba,b,Ca,b, respectively). However, P50.2 (Family 3), although showing no difference with its transportation control (Figure 8Aa), demonstrated increased conductance compared to the general, pooled, control (Figure 8Ab). There was also a family (Family 4 with P52.1) in which the Piezo1 mutation (c.7483_7488dupCTGGAG p.2495_2496dupLeuGlu) was associated with a decreased conductance compared both to the transportation and to the general, pooled, control (Figure 8Ba,b, respectively). Both Family 3 and Family 4 did not show a change in their capacitance compared to their transportation or to the general control.

### Enzymopathies

**FIGURE 9:**
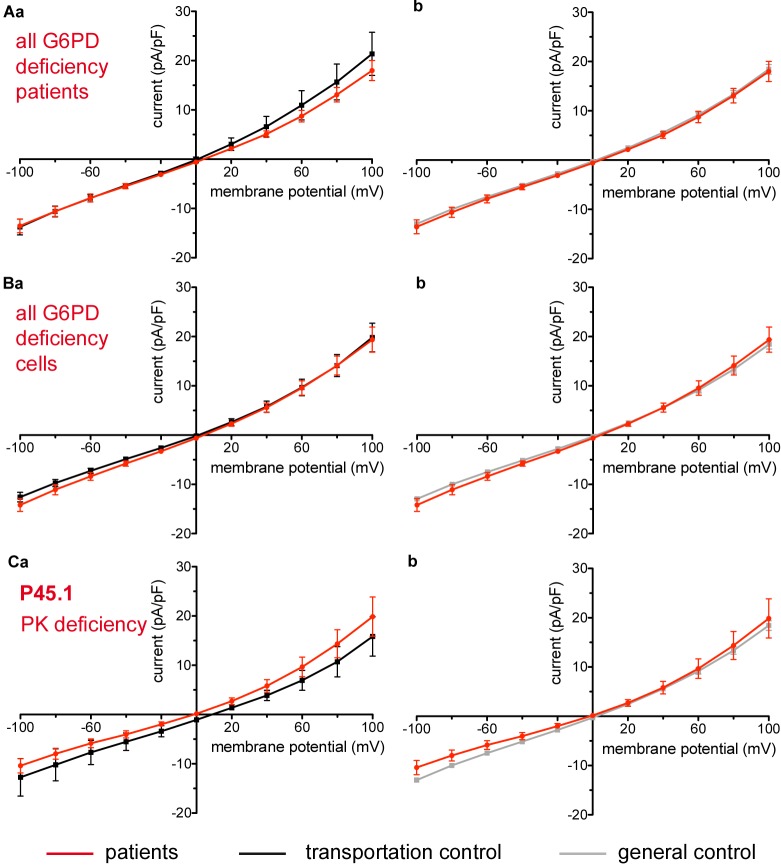
Whole-cell recordings of ion currents from RBCs of healthy donors and patients with enzymopathies (glucose-6-phosphate dehydrogenase deficiency and pyruvate kinase deficiency). Compared are the I/V-curve of all glucose-6-phosphate dehydrogenase deficiency patients (*N* = 3) with the I/V curve of their own transportation controls (*N* = 3) **(Aa)** and with the I/V curve of all healthy subjects delivered throughout the study (*N* = 27) **(Ab)**, where *N* denotes the number of healthy subjects or patients. Capacitance of the three patients P40.1, P41.1, P42.1 was not any different from that of the controls (0.62 pA/pF P40.1 + P41.1 + P42.1 vs. 0.68 pA/pF C40 + C41 + C42, *p* > 0.05; 0.62 pA/pF P40.1 + P41.1 + P42.1 vs. 0.69 pA/pF general control, *p* > 0.05). Compared are the I/V-curve of all glucose-6-phosphate dehydrogenase deficiency patients cells (*n* = 27) with the I/V curve of all transportation control cells (*n* = 22) **(Ba)** as well as with a general control (*n* = 175) **(Bb)**, where *n* denotes the number of cells. Compared are the I/V curves of P45.1 (“PK” below the patient number stands for pyruvate kinase deficiency) (*n* = 15) with its own transportation control C45 (*n* = 5) **(Ca)** as well as with a general control (*n* = 175) **(Cb)**. No changes were observed in capacitance either with the transportation (0.72 pA/pF P45.1 vs. 0.68 pA/pF C45; *p* > 0.05) or with the general control (0.72 pA/pF P45.1 vs. 0.69 pA/pF general control; *p* > 0.05). Currents were elicited by voltage steps from –100 to 100 mV for 500 ms in 20 mV increments at *V*_h_ = –30 mV. Data are expressed as mean current density ± SEMs.

**FIGURE 10:**
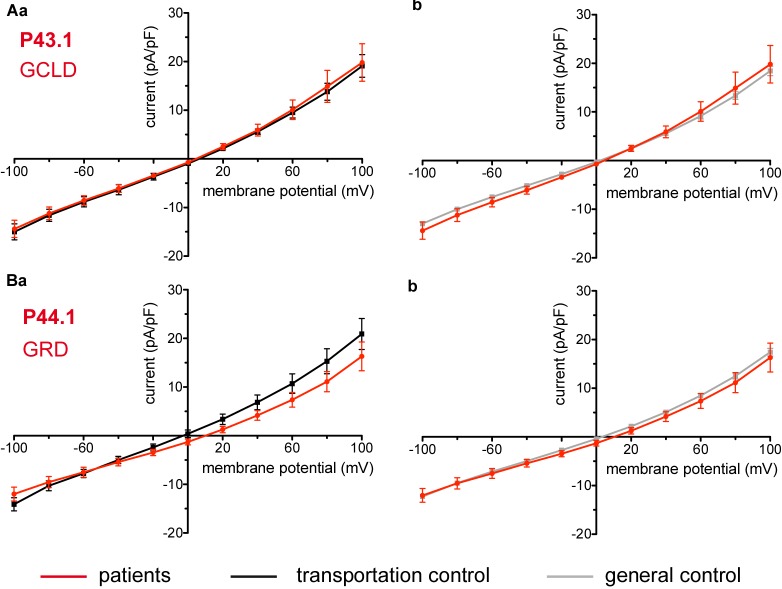
Whole-cell recordings of ion currents from RBCs of healthy donors and patients with enzymopathies. Compared are the I/V curves of P43.1 (GCLD below the patient number stands for glutamate-cysteine ligase deficiency) (*n* = 6) with its own transportation control C43 (*n* = 5) **(Aa)** as well as with a general control (*n* = 175) **(Ab)**. No changes were observed in capacitance either with the transportation (0.64 pA/pF P43.1 vs. 0.65 pA/pF C43; *p* > 0.05) or with the general control (0.64 pA/pF P43.1 vs. 0.69 pA/pF general control; *p* > 0.05). Compared are the I/V curves of P44.1 (GRD below the patient number stands for glutathione reductase deficiency) (*n* = 9) with its own transportation control C44 (*n* = 5) **(Ba)** as well as with a general control (*n* = 175) **(Bb)**, where *n* denotes the number of cells. No changes were observed in capacitance either with the transportation (0.76 pA/pF P44.1 vs. 0.65pA/pF C44; *p* > 0.05) or with the general control (0.76 pA/pF P44.1 vs. 0.69 pA/pF general control; *p* > 0.05). Currents were elicited by voltage steps from -100 to 100 mV for 500 ms in 20 mV increments at *V*_h_ = -30 mV. Data are expressed as mean current density ± SEMs.

Considered were 6 patients with enzymatic disorders as follows: 3 patients with glucose-6-phosphate dehydrogenase deficiency (P40.1, P41.1, and P42.1), 1 patient with pyruvate kinase deficiency (P45.1), 1 patient with glutamate-cysteine ligase deficiency (P43.1) and 1 patient with glutathione reductase deficiency (P44.1). None of the patients showed a difference in their membrane conductance or capacitance compared to a general or their own transportation control (Figure 9, 10).

### Beta-Thalassemia

**FIGURE 11:**
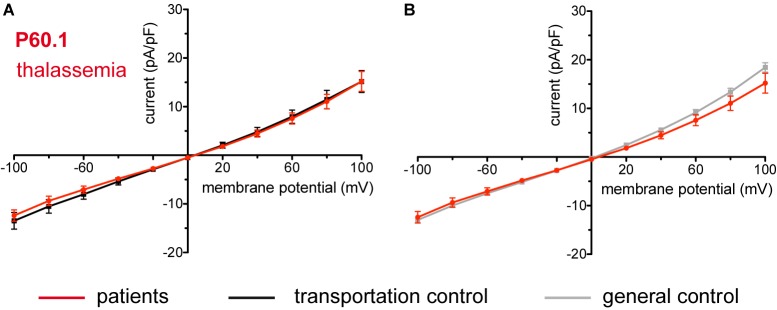
Whole-cell recordings of ion currents from RBCs of healthy donors and a patient with β-thalassemia. Compared are the I/V curves of P60.1 (*n* = 12) with its own transportation control C60 (*n* = 5) **(A)** as well as with a general control (*n* = 175) **(B)**. No changes were observed in capacitance either with the transportation (0.72 pA/pF P60.1 vs. 0.64 pA/pF C60; *p* > 0.05) or with the general control (0.72 pA/pF P60.1 vs. 0.69 pA/pF general control; *p* > 0.05). Currents were elicited by voltage steps from –100 to 100 mV for 500 ms in 20 mV increments at *V*_h_ = –30 mV. Data are expressed as mean current density ± SEMs.

The patient (P60.1) with β-thalassemia showed no difference in its membrane conductance or membrane capacitance either compared to a general or to its own, transportation, control (Figure 11).

## Discussion

### Hereditary Spherocytosis

Electrophysiological measurements revealed additional new characteristics for HS and confirmed the heterogeneity of the disease showing that changes in membrane conductance are not an overall feature of the disease but depend on the particular, specific mutation (Huisjes et al., unpublished).

Interesting is that out of the many patients with SPTA1 mutations it is the two patients carrying a *SPTA1* α^LELY^ allele that show a change in their membrane conductance: an increase in their inward current. This holds true for P18.1 vs. its own transportation control and vs. the general control of 175 cells and also for patient P19.1 vs. the pooled control. Although not reaching statistical significance the inward current of P19.1 compared to its own transportation control is also increased. Evident are the large variations within the cells of the patients for both patient P18.1 and P19.1 but especially for P19.1. This might explain why the difference in the inward current between P19.1 and its transportation control does not reach a statistical significance.

Allele α^LELY^ is a common polymorphic allele and its presence in humans is by itself asymptomatic. The α^LELY^ allele, however, plays the role of an exacerbating factor when it occurs in trans to an α-spectrin mutation resulting in a disastrously weak spectrin network (Viel and Branton, 1996; Iolascon et al., 2003). This is because, due to their reduced ability to form dimers, α chains from α^LELY^ alleles are underrepresented in the mature RBC cytoskeleton. Accordingly underrepresented are any spectrin mutations found on the same allele and in turn overrepresented if found on the opposite allele (Wilmotte et al., 1997). How a destabilized cytoskeleton might have an effect on membrane conductance is a subject of speculations but as the RBC membrane has little structural integrity without the support of an intact and steady protein scaffold below, it might be that conformational changes influence the proper functioning of channels and lead to increased membrane conductance.

Noteworthy, in many cases, aggravating conditions such as an α^LELY^ allele or a superimposed erythrocytic defect lead to an enhanced membrane conductance and a leaky cell. Thus P11.1 with a mutation in β-spectrin as well as a mutation in RHAG and P22.1 with a mutation in α-spectrin as well as being a pyruvate kinase deficiency and a thalassemia carrier show an increase both in their inward and outward current. While in the case of P22.1 such an increase cannot be straightforwardly explained as neither thalassemia nor pyruvate kinase deficiency alone give any change in conductance, P11.1 is particularly interesting. The Rh-associated glycoprotein (RhAG) coded by the RHAG gene, together with the RhD and RhCcEe proteins, is a major component of the Rh blood group system. It is essential for assembly of the Rh protein complex in the RBC membrane and for expression of the Rh antigens (Avent and Reid, 2000). The exact function of RhAG is not completely understood but it is suggested to be involved in RBC gas exchange as it promotes transmembrane NH_3_ transport (additionally NH_4_^+^) (Bakouh et al., 2006) as well as facilitates CO_2_ membrane permeation (Endeward et al., 2008). More interesting is, however, that RhAG can act as a pore for monovalent cations (Na^+^, K^+^, and Li^+^) and RHAG point mutations leading to Ile61Arg and Phe65Ser substitutions result in massively increased permeabilities for K^+^ and Na^+^ and to overhydrated stomatocytosis (Bruce et al., 2009). Expression of the mutated RhAGs in xenopus oocytes confirms the large monovalent leaks imposed by the mutations and modeling studies correlate those leaks with possibly widening the pore structures permitting passive diffusion of Na^+^ and K^+^ (Bruce et al., 2009). Mutation c.808G > A in P11.1 with a substitution Val270Ile residing in the 5th endoloop of RhAG (Huang et al., 1999) although not linked to overhydrated stomatocytosis relates to the Rh^null^ syndrome accordingly characterized by varying degrees of chronic haemolytic anemia and spherostomatocytosis (Nash and Shojania, 1987). Without knowing the mechanism that might relate mutation RHAG c.808G > A (or the complex defect c.808G > A+SPTB c.154delC) to changes in the RBC membrane conductance, P11.1 has been described with having less K^+^ in the plasma than its transportation control yet more than a non-transported normal control and a much increased activity of its Na^+^/K^+^ pump (Huisjes et al., unpublished). Thus, it could well be that a possibly increased K^+^ leak underlined by the detected increased membrane conductance triggers compensatory changes, namely enhanced ion pumping that might explain the partially compensated K^+^ leak (i.e., that K^+^ in the plasma of the patient is less than K^+^ in the plasma of its transportation control). This is in line with observations that haemolytic diseases showing increased non-Na^+^/K^+^ pump and non-NaK2Cl cotransport, K^+^ fluxes [ouabain- (an inhibitor of the Na^+^/K^+^ pump) and bumetanide-(an inhibitor of the NaK2Cl cotransport) resistant K^+^ fluxes] are accompanied by increased Na^+^/K^+^ pump fluxes (ouabain- sensitive fluxes) (Stewart, 2004).

Not always, however, a resulting changed membrane conductance is manifested as an increase in current. A patient (P12.1) with a mutation in band 3 protein shows a decrease in current and, although counterintuitive, also accompanied by a significant loss of K^+^ from the RBC.

The major function of band 3 protein is of an anion transporter exchanging a bicarbonate for a chloride ion across the RBC plasma membrane thus ensuring efficient removal of CO_2_ from tissues (Guyton and Hall, 2006). However, data in the literature show that point mutations resulting in single amino acid substitutions cause Na^+^ and K^+^ leaks with anion transport activity either maintained or abolished depending on the mutation (Bruce et al., 1993, 2005; Salhany et al., 1995; Stewart et al., 2010, 2011). Those mutations causing predominantly stomatocytosis but also spherocytosis (Arakawa et al., 2015) have been suggested to induce monovalent cation leaks in one of 3 possible ways: (i) converting the anion exchanger in a non-selective cation conductor, (ii) inducing cation conductance in a still functioning exchanger and (iii) causing the anion exchanger to stimulate endogenous cation transporters (Badens and Guizouarn, 2016). The latter has been shown in heterologous expression systems (Stewart et al., 2010) as well as in RBCs (H734R) (Bogdanova et al., 2009). It could indeed be that band 3 protein is engaged in complex interactions modulating cation permeability pathways (both channels and transporters) and that mutations changing its conformation or its availability in the membrane might lead to multifaceted effects either increasing or decreasing membrane conductance (both of which with the detrimental result of disturbing RBC ion homeostasis). In line is a study showing kidney band 3 protein interaction with nephrin (Wu et al., 2010). The intracellular domain of nephrin interacts with TRPC6, believed to be present in erythrocytes (Foller et al., 2008; Danielczok J. et al., 2017) suggesting a possible functional link between band 3 protein and TRPC6 in erythrocytes as well. While no changes in cation channel activity (not an increase either, regardless of the reported substantially elevated cation leak) have been detected in band 3 protein R730C RBCs [as measured by on-cell patch-clamp, (Stewart et al., 2011)] as well as in band 3 protein H734R RBCs [as judged by membrane potential changes, (Bogdanova et al., 2009)], it could be that each mutation alters in a different way endogenous permeability pathways.

Regarding mutation Ile783Asn of patient P12.1, it is not in the cytoplasmic half of the core domain of band 3 protein, where most of the mutations causing stomatocytosis are (Arakawa et al., 2015), yet it is very close to and in the same transmembrane domain (TM 12) as another mutation, namely Gly796Arg, also triggering stomatocytosis (Arakawa et al., 2015). According to (Huisjes et al., unpublished) mutation Ile783Asn is accompanied by increased Na^+^-K^+^-ATPase activity, a referral to stomatocytosis, yet by an osmoscan curve with a typical HS pattern and extremely low eosin-5-maleimide (EMA) staining (61%), likely reflecting strongly reduced copy numbers of band 3 protein, a referral to spherocytosis. (EMA-binding on RBCs involves the ε-NH group of lysine at position 430 from band 3 protein (Nicolas et al., 2003) and is experimentally found to correlate with band 3 protein expression on RBCs (Huisjes et al., 2018). Thus the question whether it is the unavailability of band 3 protein or a possible structural and conformational change that causes the decreased outward current, remains open.

In our study, as outlined in the Results section, we have compared the currents measured from the RBCs of our patients once with their transportation control, i.e., currents measured from the RBCs of a healthy subject, whose blood was delivered together with the blood of the patient, and once with a general control, i.e., currents measured from the cells of all healthy subjects delivered throughout the study. Inevitably a question comes up, especially when there are differences in the comparisons with the two controls, which is the more appropriate one. Whereas, undoubtedly, considering the transportation control, allows us to take into account the particular transportation conditions such as temperature, vibration intensity and shipment duration, it has limitations. Such limitations are the low number of measured cells but mostly the fact that the control subject, although judged healthy, might not be a representative control. Thus in the case with C17, the control for patients P17.1 and P17.2, the averaged capacitance of C17 RBCs (0.53 pA/pF) is statistically significantly smaller than the averaged capacitance of the RBCs of the general control (0.69 pA/pF). This in turn results in an increased current density (current divided by capacitance) for the transportation control which might explain the observed difference of P17.1 and P17.2 compared to C17 but not to the general control (Figure 4B,C).

With C10, the control of P10.1, the situation is even more extreme, as, even though the averaged capacitance of the RBCs of the transportation control is higher compared to the averaged capacitance of the general control, which results in a lower current density, the current density still remains much higher than the one of the general control. The appearance of the I/V curve is also very different with the reversal potential being much shifted to the more negative values compared to the general control (Figure 5Bc). The above mentioned limitations could be avoided by considering the general control which, due to the high number of cells, is balancing (smoothing out) the effect of a healthy but unrepresentative subject and is close to an ‘ideal’ control. At the same time what is an advantage of the general control is simultaneously a disadvantage as it ‘balances’ also the specific transportation effects on the samples. A way out of accidentally coming across a non-representative healthy subject is using the blood of several healthy subjects as a transportation control or, even better, the blood of several healthy relatives. A further problem, however, is that transportation could have different effects on the patient and on the control. This problem could be avoided by avoiding transportation itself, whenever possible.

### Hereditary Xerocytosis

Piezo 1 is a mechanically activated cation channel (Coste et al., 2010, 2012), which is permeable to monovalent cations (*P*_K_ > *P*_Cs_ ≅ *P*_Na_ > *P*_Li_) and to most divalent cations like Ba^2+^, Ca^2+^, and Mg^2+^, but not Mn^2+^ (Gnanasambandam et al., 2015). Expressed in many tissues like kidney, lung and urinary bladder (Coste et al., 2010; Miyamoto et al., 2014), Piezo 1 has been detected in the plasma membrane of RBCs, e.g., by mass spectroscopy and immunologically (Zarychanski et al., 2012; Andolfo et al., 2013; Kaestner and Egée, 2018). A major role of Piezo1 channels in RBCs is in volume regulation and mutations in the channel have been linked to HX, a dominantly inherited haemolytic anemia, characterized by decreased K^+^ and increased Na^+^ RBCs content, as well as dehydration resulting in increased mean corpuscular hemoglobin concentrations (MCHC), a leftward shift of the osmotic gradient ektacytometry curve and increased osmotic resistance of the RBCs (Gallagher, 2013; Shmukler et al., 2014; Glogowska and Gallagher, 2015; Andolfo et al., 2016). Disease clinical manifestations are variable and may include mild to moderate haemolysis, perinatal edema and non-immune hydrops fetalis that spontaneously resolve, thrombosis, pseudohyperkalemia and sometimes severe iron overload in the course of the disease (Gallagher, 2013; Glogowska and Gallagher, 2015; Andolfo et al., 2016). Out of the four mutations in our study [R2456H (P51.3 and P51.4), R2088G (P53.3 and P53.2), C426R (P50.2), and E2496ELE (P52.1)], three (R2456H, R2088G, and E2496ELE) are known and have been extensively characterized including expression of the mutant Piezo1 channel in heterologous systems and characterization of the channel activity (Zarychanski et al., 2012; Albuisson et al., 2013; Andolfo et al., 2013; Bae et al., 2013; Sandberg et al., 2014; Shmukler et al., 2014; Glogowska et al., 2017). Two of the mutations, R2456H and E2496ELE, among the most common ones found in typical HX patients (Zarychanski et al., 2012; Albuisson et al., 2013; Andolfo et al., 2013; Shmukler et al., 2014), are in the C-terminal region, part of the pore module of Piezo1 (Coste et al., 2015; Ge et al., 2015). Those mutations do not alter the sensitivity of the channel to mechanical stimulation but cause considerable increase in the inactivation time constant thus giving rise to an increased channel activity in response to a given mechanical stimulus (Albuisson et al., 2013; Glogowska et al., 2017). Could an increased channel activity lasting only for the duration of a short mechanical stimulus explain the ion disbalance observed in HX? A possible answer is that during their circulation in the vascular system RBCs are under constant mechanical stress squeezing in capillaries and in the tiny slits of the spleen undergoing numerous rounds of mechanical stimulation with Piezo1 activation (Danielczok J.G. et al., 2017) and, in the case of Piezo1 prolonged inactivation, not being capable of replenishing their ions. However, it is also likely that slowing of inactivation could potentially cause a slight increase in a basal Piezo1 activity (independent of mechanical stimulation) (Albuisson et al., 2013). This is supported by the study of (Andolfo et al., 2013), which demonstrates spontaneous ion channel activity in R2456H patient RBCs, blocked by GsMTx-4 and not observed in healthy cells. In addition are the on-cell patch recordings from the RBCs of another R2456H patient revealing once again an increased cation channel activity (independent of mechanical stimulation) without information on the identity of the channel (Shmukler et al., 2014). Consistent with Piezo 1 increased activity having direct or secondary effects on membrane conductance is the observed decrease in conductance in P52.1 (E2496ELE) RBCs. Also in P51.3 and P51.4 (R2456H) and P52.1 (E2496ELE) a rightward shift of the I/V curve is observed (Figure 7Bb, 8Bb) indicative of an upregulation/downregulation of a channel/channels. Whether the shift is pertinent to the mutations though is not explicitly clear as such a shift is not detected when patients’ currents are compared with their transportation controls (Figure 7Ba, 8Ba). Concerning mutation C426R (P50.2) which shows an increase in current only with the general control but not with its transportation control we tend to disregard the difference shown with the general control as the transportation control was taken from a genetically related healthy subject and it indeed shows a remarkable similarity with the I/V curve of the patient (Figure 8Aa).

### Enzymopathies and Beta-Thalassemia

Metabolic enzyme deficiencies (glucose-6-phosphate dehydrogenase deficiency, pyruvate kinase deficiency, glutamate-cysteine ligase deficiency and glutathione reductase deficiency) as well as β-thalassemia are not accompanied by changes in membrane conductance. The former is consistent with the lack of changes also in the K^+^ and Na^+^ content of 11 patients with congenital non-spherocytic haemolytic anemia including pyruvate kinase deficiency (Vives Corrons and Besson, 2001).

## Conclusion

Trying to summarize and come up with a common channel increased activity/dysfunction or just an effect (decrease or increase in conductance) accompanying RBCs ion disbalance disorders, we stumble upon a great variability in results. Such a variability is of course reflecting the many triggers of the disbalance and for sure not lessened by the fact that not only between different mutations but also among members of a family with the same mutation there are distinct differences starting from the severity of the disease and extending down to the cellular level. Such a summary is even harder and certainly not helped by the great variability in healthy subjects reflected in control membrane conductance measurements as seen in our study. And last but not least answers are yet heavier without so far clearly knowing how an increased Na^+^ and a decreased K^+^ can lead to overhydration as in OHSt or to dehydration as in HX. Nevertheless based on our study we conclude that changes in conductance are incurred by certain α-spectrin [c.2755G > T (p.Glu919) and c.678G > A p.(Glu227fs) whenever an α^LELY^ allele is present], band 3 protein [c.2348T > A p.(Ile783Asn)] and Piezo1 (c.7483_7488dupCTGGAG p.2495_2496dupLeuGlu) mutations as a difference is observed with both the general and the transportation control. Identification of the channel/channels that underlie the changed conductances demands future studies.

## Ethics Statement

Patients’ data were handled anonymously as outlined in the ethics agreements. These agreements were approved by the Medical Ethical Research Board (MERB) of the University Medical Center Utrecht, Netherlands (UMCU) under reference code 15/426M “Disturbed ion homeostasis in hereditary hemolytic anemia” and by the Ethical Committee of Clinical Investigations of Hospital Clinic, Spain (IDIBAPS) under reference code 2013/8436.

## Author Contributions

All authors listed have made a substantial, direct and intellectual contribution to the work, and approved it for publication.

## Conflict of Interest Statement

The authors declare that the research was conducted in the absence of any commercial or financial relationships that could be construed as a potential conflict of interest.

## References

[B1] Albuisson J. Murthy S. E. Bandell M. Coste B. Louis-Dit-Picard H. Mathur J. (2013). Dehydrated hereditary stomatocytosis linked to gain-of-function mutations in mechanically activated PIEZO1 ion channels. *Nat. Commun.* 4:1884. 10.1038/ncomms2899 23695678 PMC3674779

[B2] Andolfo I. Alper S. L. De Franceschi L. Auriemma C. Russo R. De Falco L. (2013). Multiple clinical forms of dehydrated hereditary stomatocytosis arise from mutations in PIEZO1. *Blood* 121 3925–3935. 10.1182/blood-2013-02-482489 23479567

[B3] Andolfo I. Russo R. Gambale A. Iolascon A. (2016). New insights on hereditary erythrocyte membrane defects. *Haematologica* 101 1284–1294. 10.3324/haematol.2016.142463 27756835 PMC5394881

[B4] Arakawa T. Kobayashi-Yurugi T. Alguel Y. Iwanari H. Hatae H. Iwata M. (2015). Crystal structure of the anion exchanger domain of human erythrocyte band 3. *Science* 350 680–684. 10.1126/science.↑4335 26542571

[B5] Avent N. D. Reid M. E. (2000). The Rh blood group system: a review. *Blood* 95 375–387. 10627438

[B6] Badens C. Guizouarn H. (2016). Advances in understanding the pathogenesis of the red cell volume disorders. *Br. J. Haematol.* 174 674–685. 10.1111/bjh.14197 27353637

[B7] Bae C. Gnanasambandam R. Nicolai C. Sachs F. Gottlieb P. A. (2013). Xerocytosis is caused by mutations that alter the kinetics of the mechanosensitive channel PIEZO1. *Proc. Natl. Acad. Sci. U.S.A.* 110 E1162–E1168. 10.1073/pnas.1219777110 23487776 PMC3606986

[B8] Bakouh N. Benjelloun F. Cherif-Zahar B. Planelles G. (2006). The challenge of understanding ammonium homeostasis and the role of the Rh glycoproteins. *Transfus. Clin. Biol.* 13 139–146. 10.1016/j.tracli.2006.02.008 16564724

[B9] Bogdanova A. Y. Goede J. S. Weiss E. Bogdanov N. Bennekou P. Bernhardt I. (2009). Cryohydrocytosis: increased activity of cation carriers in red cells from a patient with a band 3 mutation. *Haematologica* 95 189–198. 10.3324/haematol.2009.010215 20015879 PMC2817020

[B10] Bruce L. J. Guizouarn H. Burton N. M. Gabillat N. Poole J. Flatt J. F. (2009). The monovalent cation leak in overhydrated stomatocytic red blood cells results from amino acid substitutions in the Rh-associated glycoprotein. *Blood* 113 1350–1357. 10.1182/blood-2008-07-171140 18931342

[B11] Bruce L. J. Kay M. M. Lawrence C. Tanner M. J. (1993). Band 3 HT, a human red-cell variant associated with acanthocytosis and increased anion transport, carries the mutation Pro-868→Leu in the membrane domain of band 3. *Biochem. J.* 293 317–320. 10.1042/bj2930317 8343110 PMC1134360

[B12] Bruce L. J. L. Robinson H. C. H. Guizouarn H. H. Borgese F. F. Harrison P. P. King M.-J. M. (2005). Monovalent cation leaks in human red cells caused by single amino-acid substitutions in the transport domain of the band 3 chloride-bicarbonate exchanger, AE1. *Nat. Genet.* 37 1258–1263. 10.1038/ng1656 16227998

[B13] Cao A. Galanello R. (2010). Beta-thalassemia. *Genet. Med.* 12 61–76. 10.1097/GIM.0b013e3181cd68ed 20098328

[B14] Coste B. Mathur J. Schmidt M. Earley T. J. Ranade S. Petrus M. J. (2010). Piezo1 and Piezo2 are essential components of distinct mechanically activated cation channels. *Science* 330 55–60. 10.1126/science.1193270 20813920 PMC3062430

[B15] Coste B. Murthy S. E. Mathur J. Schmidt M. Mechioukhi Y. Delmas P. (2015). Piezo1 ion channel pore properties are dictated by C-terminal region. *Nat. Commun.* 6:7223. 10.1038/ncomms8223 26008989 PMC4445471

[B16] Coste B. Xiao B. Santos J. S. Syeda R. Grandl J. Spencer K. S. (2012). Piezo proteins are pore-forming subunits of mechanically activated channels. *Nature* 483 176–181. 10.1038/nature10812 22343900 PMC3297710

[B17] Danielczok J. G. Terriac E. Hertz L. Petkova-Kirova P. Lautenschläger F. Laschke M. W. (2017). Red blood cell passage of small capillaries is associated with transient Ca2+-mediated adaptations. *Front. Physiol.* 8:979. 10.3389/fphys.2017.00979 29259557 PMC5723316

[B18] Danielczok J. Hertz L. Ruppenthal S. Kaiser E. Petkova-Kirova P. Bogdanova A. (2017). Does erythropoietin regulate TRPC channels in red blood cells? *Cell. Physiol. Biochem.* 41 1219–1228. 10.1159/000464384 28268218

[B19] Dhaliwal G. Cornett P. A. Tierney L. M. (2004). Hemolytic anemia. *Am. Fam. Physician* 69 2599–2606. 15202694

[B20] Endeward V. Cartron J.-P. Ripoche P. Gros G. (2008). RhAG protein of the Rhesus complex is a CO2 channel in the human red cell membrane. *FASEB J.* 22 64–73. 10.1096/fj.07-9097com 17712059

[B21] Fermo E. Bogdanova A. Petkova-Kirova P. Zaninoni A. Marcello A. P. Makhro A. (2017). “Gardos Channelopathy”: a variant of hereditary stomatocytosis with complex molecular regulation. *Sci. Rep.* 7:1744. 10.1038/s41598-017-01591-w 28496185 PMC5431847

[B22] Foller M. Kasinathan R. S. Koka S. Lang C. Shumilina E. V. Birnbaumer L. (2008). TRPC6 contributes to the Ca(2+) leak of human erythrocytes. *Cell. Physiol. Biochem.* 21 183–192. 10.1159/000113760 18209485

[B23] Gallagher P. G. (2013). Disorders of red cell volume regulation. *Curr. Opin. Hematol.* 20 201–207. 10.1097/MOH.0b013e32835f6870 23519154

[B24] Ge J. Li W. Zhao Q. Li N. Chen M. Zhi P. (2015). Architecture of the mammalian mechanosensitive Piezo1 channel. *Nature* 527 64–69. 10.1038/nature15247 26390154

[B25] Glogowska E. Gallagher P. G. (2015). Disorders of erythrocyte volume homeostasis. *Int. J. Lab. Hematol.* 37(Suppl. 1), 85–91. 10.1111/ijlh.12357 25976965 PMC4435826

[B26] Glogowska E. Schneider E. R. Maksimova Y. Schulz V. P. Lezon-Geyda K. Wu J. (2017). Novel mechanisms of PIEZO1 dysfunction in hereditary xerocytosis. *Blood* 130 1845–1856. 10.1182/blood-2017-05-786004 28716860 PMC5649553

[B27] Gnanasambandam R. Bae C. Gottlieb P. A. Sachs F. (2015). Ionic selectivity and permeation properties of human PIEZO1 channels. *PLoS One* 10:e0125503. 10.1371/journal.pone.0125503 25955826 PMC4425559

[B28] Guyton A. C. Hall J. E. (2006). *Medical Physiology.* Amsterdam: Elsevier Saunders.

[B29] Hertz L. Huisjes R. Llaudet-Planas E. Petkova-Kirova P. Makhro A. Danielczok J. G. (2017). Is increased intracellular calcium in red blood cells a common component in the molecular mechanism causing anemia? *Front. Physiol.* 8:673. 10.3389/fphys.2017.00673 28932200 PMC5592231

[B30] Huang C. H. Cheng G. Liu Z. Chen Y. Reid M. E. Halverson G. (1999). Molecular basis for Rh(null) syndrome: identification of three new missense mutations in the Rh50 glycoprotein gene. *Am. J. Hematol.* 62 25–32. 10.1002/(SICI)1096-8652(199909)62:1<25::AID-AJH5>3.0.CO;2-K 10467273

[B31] Huisjes R. Satchwell T. J. Verhagen L. P. Schiffelers R. M. van Solinge W. W. Toye A. M. (2018). Quantitative measurement of red cell surface protein expression reveals new biomarkers for hereditary spherocytosis. *Int. J. Lab. Hematol.* 40 e74–e77. 10.1111/ijlh.12841 29746727

[B32] Iolascon A. Perrotta S. Stewart G. W. (2003). Red blood cell membrane defects. *Rev. Clin. Exp. Hematol.* 7 22–56. 14692233

[B33] Kaestner L. Egée S. (2018). Commentary: voltage gating of mechanosensitive PIEZO channels. *Front. Physiol.* 9:1565. 10.3389/fphys.2018.01565 30524293 PMC6256199

[B34] Luzzatto L. Nannelli C. Notaro R. (2016). Glucose-6-phosphate dehydrogenase deficiency. *Hematol. Oncol. Clin. North Am.* 30 373–393. 10.1016/j.hoc.2015.11.006 27040960

[B35] Makhro A. Huisjes R. Verhagen L. P. Mañú Pereira M. D. M. Llaudet-Planas E. Petkova-Kirova P. (2016). Red cell properties after different modes of blood transportation. *Front. Physiol.* 7:288. 10.3389/fphys.2016.00288 27471472 PMC4945647

[B36] Miyamoto T. Mochizuki T. Nakagomi H. Kira S. Watanabe M. Takayama Y. (2014). Functional role for Piezo1 in stretch-evoked Ca^2+^ influx and ATP release in urothelial cell cultures. *J. Biol. Chem.* 289 16565–16575. 10.1074/jbc.M113.528638 24759099 PMC4047422

[B37] Nash R. Shojania A. M. (1987). Hematological aspect of Rh deficiency syndrome: a case report and a review of the literature. *Am. J. Hematol.* 24 267–275. 10.1002/ajh.2830240306 3103426

[B38] Nicolas V. Le Van Kim C. Gane P. Birkenmeier C. Cartron J.-P. Colin Y. (2003). Rh-RhAG/ankyrin-R, a new interaction site between the membrane bilayer and the red cell skeleton, is impaired by Rh(null)-associated mutation. *J. Biol. Chem.* 278 25526–25533. 10.1074/jbc.M302816200 12719424

[B39] Petkova-Kirova P. Hertz L. Makhro A. Danielczok J. Huisjes R. Llaudet-Planas E. (2018). A previously unrecognized Ca2+-inhibited nonselective cation channel in red blood cells. *Hemasphere* 2:e146. 10.1097/HS9.0000000000000146 30887009 PMC6407795

[B40] Ristoff E. Larsson A. (1998). Patients with genetic defects in the gamma-glutamyl cycle. *Chem. Biol. Interact.* 11 113–121. 10.1016/S0009-2797(97)00155-5 9679548

[B41] Rotordam G. M. Fermo E. Becker N. Barcellini W. Brüggemann A. Fertig N. (2018). A novel gain-of-function mutation of Piezo1 is functionally affirmed in red blood cells by high-throughput patch clamp. *Haematologica* 10.3324/haematol.2018.201160 [Epub ahead of print]. 30237269 PMC6518891

[B42] Salhany J. M. Schopfer L. M. Kay M. M. Gamble D. N. Lawrence C. (1995). Differential sensitivity of stilbenedisulfonates in their reaction with band 3 HT (Pro-868– > Leu). *Proc. Natl. Acad. Sci. U.S.A.* 92 11844–11848. 10.1073/pnas.92.25.11844 8524861 PMC40499

[B43] Sandberg M. B. Nybo M. Birgens H. Frederiksen H. (2014). Hereditary xerocytosis and familial haemolysis due to mutation in the PIEZO1 gene: a simple diagnostic approach. *Int. J. Lab. Hematol.* 36 e62–e65. 10.1111/ijlh.12172 24314002

[B44] Shmukler B. E. Vandorpe D. H. Rivera A. Auerbach M. Brugnara C. Alper S. L. (2014). Dehydrated stomatocytic anemia due to the heterozygous mutation R2456H in the mechanosensitive cation channel PIEZO1: a case report. *Blood Cells Mol. Dis.* 52 53–54. 10.1016/j.bcmd.2013.07.015 23973043

[B45] Stewart A. K. Kedar P. S. Shmukler B. E. Vandorpe D. H. Hsu A. Glader B. (2011). Functional characterization and modified rescue of novel AE1 mutation R730C associated with overhydrated cation leak stomatocytosis. *Am. J. Physiol. Cell Physiol.* 300 C1034–C1046. 10.1152/ajpcell.00447.2010 21209359 PMC3093938

[B46] Stewart A. K. Vandorpe D. H. Heneghan J. F. Chebib F. Stolpe K. Akhavein A. (2010). The GPA-dependent, spherostomatocytosis mutant AE1 E758K induces GPA-independent, endogenous cation transport in amphibian oocytes. *Am. J. Physiol. Cell Physiol.* 298 C283–C297. 10.1152/ajpcell.00444.2009 19907019 PMC2822494

[B47] Stewart G. W. (2004). Hemolytic disease due to membrane ion channel disorders. *Curr. Opin. Hematol.* 11 244–250. 10.1097/01.moh.0000132240.20671.33 15314523

[B48] van Zwieten R. Verhoeven A. J. Roos D. (2014). Inborn defects in the antioxidant systems of human red blood cells. *Free Radic. Biol. Med.* 67 377–386. 10.1016/j.freeradbiomed.2013.11.022 24316370

[B49] Viel A. Branton D. (1996). Spectrin: on the path from structure to function. *Curr. Opin. Cell Biol.* 8 49–55. 10.1016/S0955-0674(96)80048-2 8791400

[B50] Vives Corrons L. Besson I. (2001). Red cell membrane Na+ transport systems in hereditary spherocytosis: relevance to understanding the increased Na+ permeability. *Ann. Haematol.* 80 535–539. 10.1007/s002770100342 11669303

[B51] Ware R. E. de Montalembert M. Tshilolo L. Abboud M. R. (2017). Sickle cell disease. *Lancet* 390 311–323. 10.1016/S0140-6736(17)30193-9 28159390

[B52] Wilmotte R. Harper S. L. Ursitti J. A. Maréchal J. Delaunay J. Speicher D. W. (1997). The exon 46-encoded sequence is essential for stability of human erythroid alpha-spectrin and heterodimer formation. *Blood* 90 4188–4196. 9354690

[B53] Wu F. Saleem M. A. Kampik N. B. Satchwell T. J. Williamson R. C. Blattner S. M. (2010). Anion exchanger 1 interacts with nephrin in podocytes. *J. Am. Soc. Nephrol.* 21 1456–1467. 10.1681/ASN.2009090921 20576809 PMC3013520

[B54] Zanella A. Fermo E. Bianchi P. Valentini G. (2005). Red cell pyruvate kinase deficiency: molecular and clinical aspects. *Br. J. Haematol.* 130 11–25. 10.1111/j.1365-2141.2005.05527.x 15982340

[B55] Zarychanski R. Schulz V. P. Houston B. L. Maksimova Y. Houston D. S. Smith B. (2012). Mutations in the mechanotransduction protein PIEZO1 are associated with hereditary xerocytosis. *Blood* 120 1908–1915. 10.1182/blood-2012-04-422253 22529292 PMC3448561

